# Evaluation of carbon:nitrogen ratio in semi-defined culture medium to tacrolimus biosynthesis by *Streptomyces tsukubaensis* and the effect on bacterial growth

**DOI:** 10.1016/j.btre.2020.e00440

**Published:** 2020-02-20

**Authors:** Jean Vinícius Moreira, Seforah Carolina Marques Silva, Marco Aurélio Cremasco

**Affiliations:** University of Campinas, School of Chemical Engineering, Department of Process Engineering, Campinas, Brazil

**Keywords:** Tacrolimus, *Streptomyces tsukubaensis*, Immunosuppressant, Submerged fermentation, Carbon:nitrogen ratio

## Abstract

Tacrolimus is an immunosuppressant produced by several *Streptomyces* species, widely used in transplantolgy and dermatology. In this report, attempts have been made to enhance tacrolimus productivity by altering the primary carbon source on culture media. In order to better understand the effects of glucose and maltose on tacrolimus biosynthesis, different carbon contents of these sugars were evaluated in the culture media, thus altering the carbon:nitrogen ratio. The highest specific production achieved on this report was 10.46 mg/g at a carbon:nitrogen ratio of 8.7:1.0 and maltose as carbon source. Using glucose as primary carbon source, the highest specific production achieved was 9.06 mg/g at a carbon:nitrogen ratio of 8.5:1.0. The range of carbon:nitrogen ratios evaluated for each sugar presented distinct behaviors. Glucose containing-media exhibited an initial linear downward trend, by the other hand, maltose containing-media exhibited an increasing tendency. Both glucose and maltose presented the highest specific production at 3 % of carbon source on media. Media containing more than 3 % of carbon content exerted carbon catabolite repression.

The range of carbon:nitogen ratios evaluated presented an increasing tendency on tacrolimus biosynthesis up to 3 % of glucose or maltose in the media, both carbon sources exerted the phenomenon of carbon catabolite repression with more than 3 % of carbon source on the media.

## Highlights

• Maltose-containing media presented a crescent tendency on tacrolimus specific production up to 3 % of maltose on media.
• Glucose-containing media presented a slight decrease on specific production from 1 % to 2 % of carbon source on media.
• Both maltose and glucose presented the best results with 3 % of primary carbon source on medium;
• Maltose-containing media supported higher tacrolimus production.
• Both maltose and glucose exerted carbon catabolite repression on media with more than 3 % of carbon source.

## 1 Introduction

**Fig. 1:**
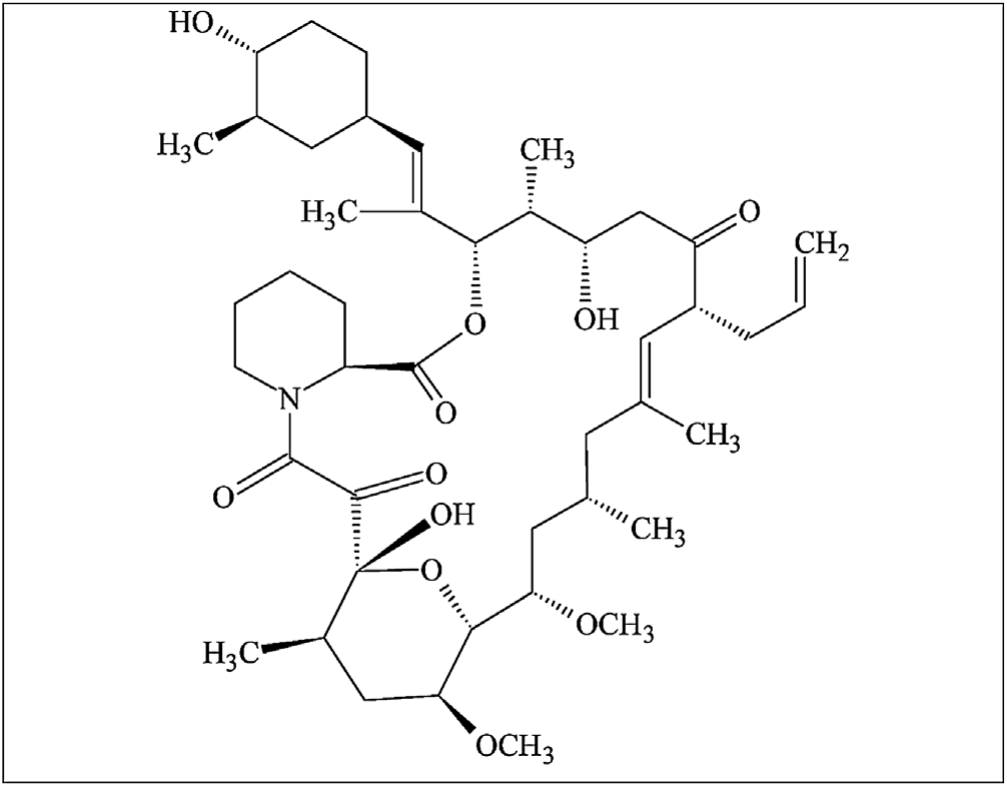
Structural formula of tacrolimus (FK 506).

Tacrolimus, also known as FK-506 or Fujimicyn, is a 23-membered polyketide macrolide with immunosuppressant activity and a molecular weight of 822 daltons. It exerts, in vitro, 10–100 times higher immunosuppressive activity than cyclosporin. Tacrolimus (Fig. 1) is produced by several *Streptomyces* species. In 1984, scientists from Fujisawa Pharmaceutical Co., isolated a tacrolimus-producing strain from the fermentation broth of a soil sample from Tsukuba, Japan, and defined it as *Streptomyces tsukubaensis* [1,2].

Tacrolimus was first reported by Kino et al. [1] and the drug launched in 1993 in Japan as immunosuppressive agent for the prevention of graft rejection after liver transplantation. After clinical trials, in 1994 tacrolimus was approved by the FDA to prevent graft rejection and launched in USA. Apart from its first uses as immunosuppressive agent, tacrolimus gained entry into the treatment for various dermatological disorders such as atopic dermatitis (AD) and psoriasis. Japan was the first country to approve topical tacrolimus application for the treatment of AD in 1999, followed by the United States in 2000 and Europe in 2001. It is also used as ointment on the treatment of vitiligo, due to its immunomodulatory characteristics and as repigmenting agent for both adults and children [[3], [4], [5], [6]].

Despite the tacrolimus clinical relevance, wide range of applications and the high importance for the pharmaceutical market, low production levels are achieved by industrial strains; thus, many researchers have studied medium optimization, genetic engineering of strains and optimization of culture conditions [[7], [8], [9]].

In order to meet the increasing demand of tacrolimus and enhance its biosynthesis, considerable effort has been spent on optimizing culture media using different carbon and nitrogen sources, amino acids and precursors. It is notable that depending on the strain and on the carbon source concentration in the media, tacrolimus productivity may increase significantly up to 2–3 folds. However, the same behavior is not observed when nitrogen source is altered. Inorganic nitrogen sources present a slightly increase in productivity. The use of some tacrolimus biosynthesis precursors may increase tacrolimus productivity up to 7 folds, depending on carbon source and the strain used [[10], [11], [12], [13]].

Complex culture media commonly contains one or more carbon sources that may be either rapidly or slowly assimilated by microorganisms Rapidly assimilated carbon sources may block or reduce the production of secondary metabolites, it resembles the phenomenon of carbon catabolite repression (CCR). Glucose, one of the most common carbon sources used on fermentative production, and also a precursor to biosynthesize secondary metabolites, is usually excellent for bacterial growth, but it may interfere with the formation of many secondary metabolites. The repression effect is not yet fully understood; however, it may be related to glucose transport and phosphorylation [12,13,14].

The *Streptomyces* genus is responsible of the production of about 70 % of clinical available antibiotics and anticancer agents; thus, it is imperative to understand CCR and its implications on the production of secondary metabolites. The importance of knowing the effects of CCR lies on defining a culture medium for production improvement by exogenous strategies before genetic engineering of the strains. Still, the complete molecular mechanism that governs CCR in genus *Streptomyces* remains unknown. The glycolytic enzyme glucose kinase (Glk), is proposed to be a key player exerting its role by interacting with transcriptional regulators [12,15].

This work aims to evaluate the effects of different carbon contents on the media, and their impact on bacterial growth and tacrolimus biosynthesis in *S. tsukubaensis* in a submerged culture in semi-defined media containing glucose and maltose as carbon sources, and corn steep liquor and soy peptone as nitrogen sources.

## 2 Materials and methods

### 2.1 Bacterial strain and seed medium

The *S. tsukubaensis* DSM – 42081 strain used in this work was obtained from the Leibniz Institute DSMZ – German Collection of Microorganisms and Cell Cultures and it is the original wild-type strain isolated in Japan [1,16].

The seed culture was prepared by adding a loopful culture of *Streptomyces tsukubaensis* in 100 mL of basal media composed of glucose 0.6 %, maltose 0.6 %, yeast extract 0.12 %, and malt extract 0.6 %. pH was adjusted to 7.2 before autoclaving. The culture was incubated at 28 °C on a rotary shaker (Shel Lab, USA) at 110 rpm for 24 h [10].

### 2.2 Characterization of the nitrogen sources

In order to accurately examine the effects of nutrients in the media, it is necessary to evaluate C and N content as total organic carbon (TOC) and total nitrogen (Kjeldahl), due to their complex characteristics. Kjeldahl nitrogen in the sample is first converted to ammonia by metal-catalyzed acid digestion. The resulting ammonia is then separated from the sample by distillation. The ammonia released is captured in a diluted sulfuric acid solution, then the ammonia concentration of the distillate is determined by colorimetric measurement. Both results will support a real determination of carbon:nitrogen ratio in each medium evaluated. The amount of carbon and nitrogen on soy peptone and corn steep liquor are exposed on Table 1, both are used as components of the basal medium for GPL and MPL media. Total nitrogen (Kjeldahl) was determined according to USP 39-NF 34, United States Pharmacopeia and the National Formulary.

**Table 1:** Carbon and Nitrogen content on complex nitrogen media.

Media	Total organic carbon (%)	Kjeldahl nitrogen (%)
Soy peptone	44.4	9.89
Corn steep liquor	12.5	1.6

### 2.3 Culture media and culture conditions

To evaluate the carbon:nitrogen (C:N) ratio on liquid medium to produce tacrolimus, a series of batch fermentations were performed. Two distinct media were studied, GPL medium with glucose as carbon source, and MPL medium with maltose as carbon source. The carbon:nitrogen ratio was modified by only altering the content of the carbon source from 1 to 4 %, and keeping the nitrogen source and the other components of culture medium as follows: soy peptone 3 %, corn steep liquor 1 %, MgSO_4_.7H_2_O 0.05 %, KH_2_PO_4_ 0.2 %, K_2_HPO_4_ 0.4 %, and CaCO_3_ 0.3 % [10].

According to Table 1 it is possible to accurately determine the C:N ratios present on studied media, and, therefore, evaluate the effects of carbon content on tacrolimus biosynthesis by *S. tsukubaensis*. The C:N ratio of each medium, listed in Table 2, was calculated by the amount of carbon and nitrogen present in glucose and maltose and added the amount of carbon and nitrogen in the corn steep liquor and soy peptone.

**Table 2:** Carbon:Nitrogen ratios evaluated for GPL and MPL media.

C:N for GPL	C:N for MPL	glucose and maltose (%)
5.9:1.0	6.1:1.0	1
7.2:1.0	7.4:1.0	2
8.5:1.0	8.7:1.0	3
9.8:1.0	10.0:1.0	4

Batch fermentations were performed in duplicates in 500-mL flasks containing 250 mL of GPL or MPL medium. A 10 % of the 24-h-old seed culture was used as inoculum. The cultures were incubated at 28 °C on a rotary shaker (Shel Lab, USA) at 130 rpm for 240 h.

### 2.4 Analytical methods

Tacrolimus production and biomass accumulation along the fermentation process were measured by taking a sample of 10 mL aseptically and periodically each 24 h. The method used and described above was based on previous works [10,11,[17], [18], [19]].

The content of tacrolimus in each 10-mL sample was extracted with an equal volume of acetone and filtered in pre-weighed Whatman no. 1 filter paper. The cells were washed three times with 100 mL of deionized water and kept at 80 °C for 24 h for drying.

The extracts were evaporated at reduced pressure until a yellow oily compound was observed. To the oily compound 3.5 mL of a binary mixture 60:40, v/v of acetonitrile and water was added. The samples were centrifuged at 2400 *g* and 4 °C for 10 min. Samples of 20 μL of the supernatant were injected in a HPLC (Shimadzu) equipped with an UV detector (SPD-20A) and monitored at 210 nm. Mobile phase was composed of acetonitrile (Scharlau, Spain. HPLC grade) and deionized water, at proportion of 60:40 (v/v) and the flow rate was maintained at 1.0 mL/min. The injections were performed in a C18 column (ThermoQuest Hyperbond 300 × 3.9 mm) kept at 60 °C.

The area of tacrolimus-related peaks in the chromatograms was used to quantify tacrolimus using a standard curve previously prepared with a stock solution (200 mg/L) of tacrolimus standard (Sigma-Aldrich) diluted in concentrations of 5, 15, 25, 50, 100, and 200 mg/L.

### 2.5 Determination of glucose and maltose consumption

Glucose consumption along the fermentation process was determined with the help of a commercial glucose oxidase kit (Bioclin, Brazil). Maltose consumption was measured by the Somogyi-Nelson method, that is based on the properties of reducing sugars when heated with alkaline copper tartrate: under these conditions the sugars reduce the copper from the cupric to the cuprous state forming cuprous oxide [20,21].

## 3 Results

### 3.1 Effect of C:N ratio variation on media containing glucose

**Fig. 2:**
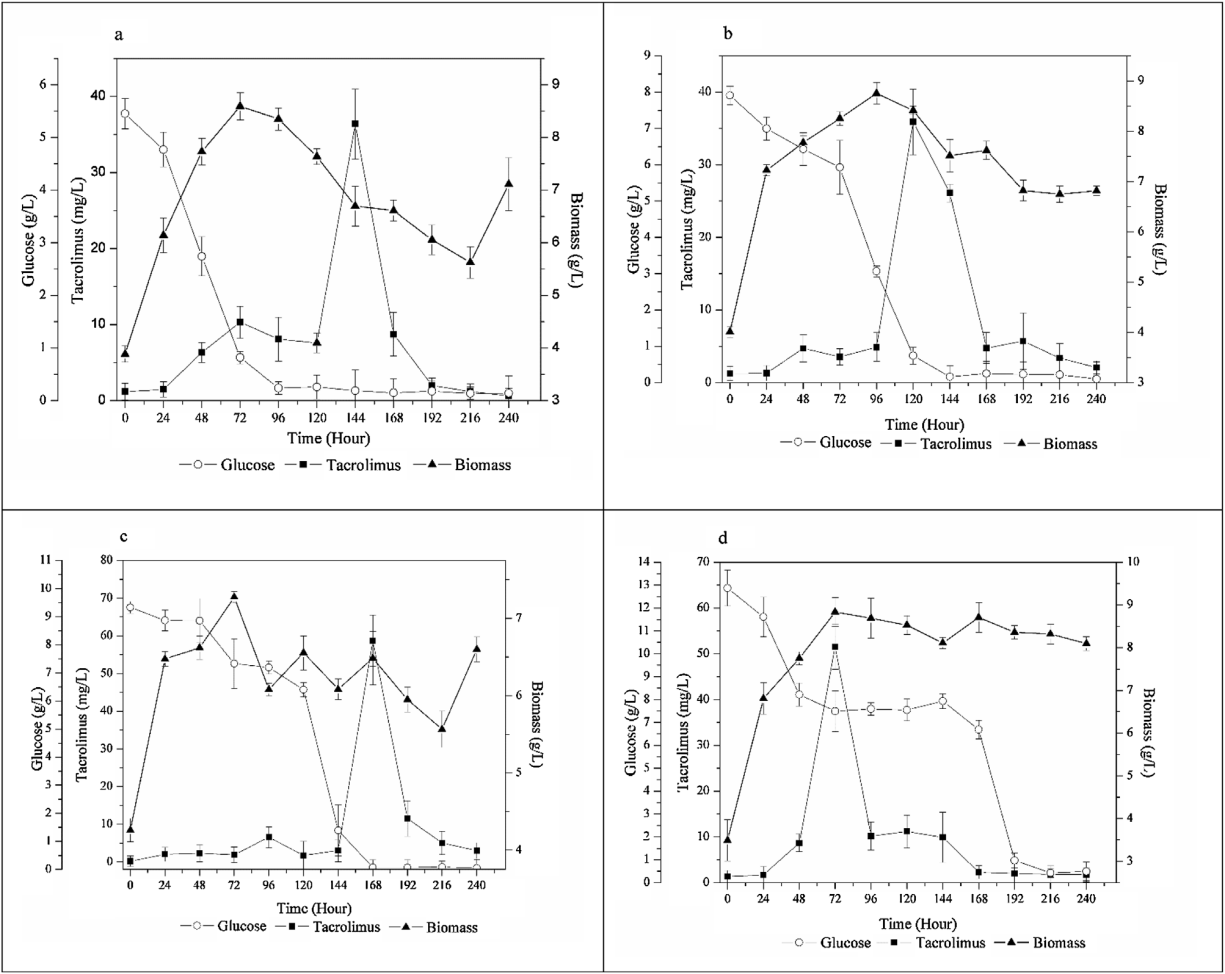
Influence of C:N ratios on tacrolimus production, bacterial growth and glucose consumption (a) C:N ratio 5.9:1.0, (b) C:N ratio 7.2:1.0, (c) C:N ratio 8.5:1.0, (d) C:N ratio 9.8:1.0.

The goal of this work was to identify and evaluate the most suitable primary carbon source and the C:N ratio for tacrolimus biosynthesis by *S. tsukubaensis*. For this purpose, glucose and maltose were studied as main carbon sources. Fig. 2 exposes the results of tacrolimus production, biomass accumulation, and glucose consumption during the fermentation process.

Fig. 2 also exposes that tacrolimus biosynthesis started after 24 h of culture, although the highest yield in each case was not achieved at the same average time, varying in each medium evaluated. The shortest time to tacrolimus maximum production was observed with medium containing 4 % of glucose (C:N ratio 9.8:1.0) exposed on Fig. 2d, after 72 h of culture, leading to 51.55 mg/L of tacrolimus and 8.84 g/L of biomass. However, the highest tacrolimus titer was obtained with 3 % of glucose on medium (C:N ratio 8.5:1.0), Fig. 2c, leading to 58.74 mg/L of tacrolimus, and 6.5 g/L of biomass. With exception of Fig. 2c that achieved lower biomass accumulation the increasing of glucose content on media did not interfere severely on cell growth. The lowest glucose content on media produced the lowest tacrolimus production, as exposed on Fig. 2a and b.

**Fig. 3:**
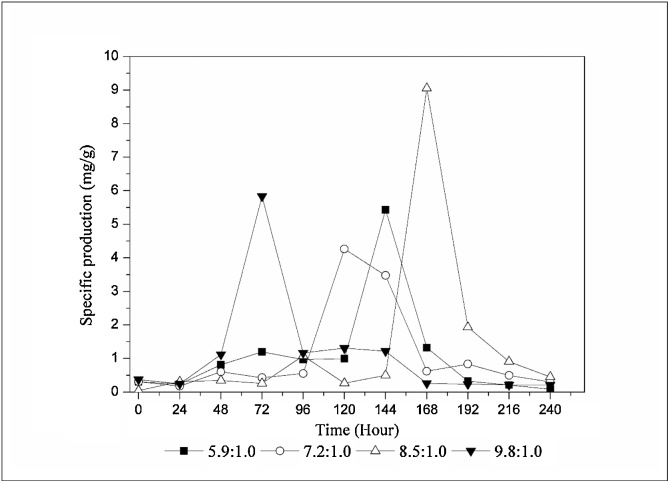
Specific production of tacrolimus with glucose.

In order to accurately evaluate the media, it was plotted the specific production of tacrolimus to each medium (Fig. 3). The highest specific production with glucose achieved on this work was 9.06 mg/g obtained with the medium containing 3 % of glucose, leading to a C:N ratio of 8.5:1.0.

Mishra and Verma [11] conducted an exploratory work on carbon and nitrogen sources in *Streptomyces* sp. The authors achieved 8.70 mg/g of tacrolimus when glucose was the primary carbon source in the medium. Martínez-Castro et al. [19] used glucose combined with starch as carbon source to produce tacrolimus with *S. tsukubaensis*, the specific production achieved by authors was about 2.8 mg/g. However, it is not possible to accurately determine the C:N ratio used on the study, due to the lack of information of carbon and nitrogen content on complex media. Wang et al. [22] used dextrin and glucose as carbon sources for tacrolimus biosynthesis by *S. tusukubaensis*, the highest specific production reported was 8.50 mg/g.

Chen et al. [13] evaluated 5 different genetically engineered strains of *S. tsukubaensis* to enhance tacrolimus productivity on glucose-containing media. Tacrolimus highest titer achieved by authors was around 120 mg/L; however, the authors did not present biomass accumulation data, which allows to evaluate specific productivity.

Besides the difference in micronutrients composition in the culture media [11,13,19,22] and the strains used between the works reported above and this work, other culture conditions might also contribute to the differences observed. For example temperature as set at 28 °C and 130 rpm; although, the temperature was the same [11,13,19,22], the rotation was set at 220 rpm [13,17,19,22] and at 200 rpm [11]. It indicates that agitation holds an important role on defining culture conditions, due to the wide range of specific productivity achieved by each author.

### 3.2 Effect of C:N ratio variation on media containing maltose

The basal medium, first reported by Turło et al. [10] and Gajzlerska et al. [23] suggested the use of 2 % of maltose as primary carbon source. However, the authors did not perform a study of the variation of carbon content on medium the authors evaluated the effect of the addition of tacrolimus precursors on tacrolimus biosynthesis and strain growth promoters.

**Fig. 4:**
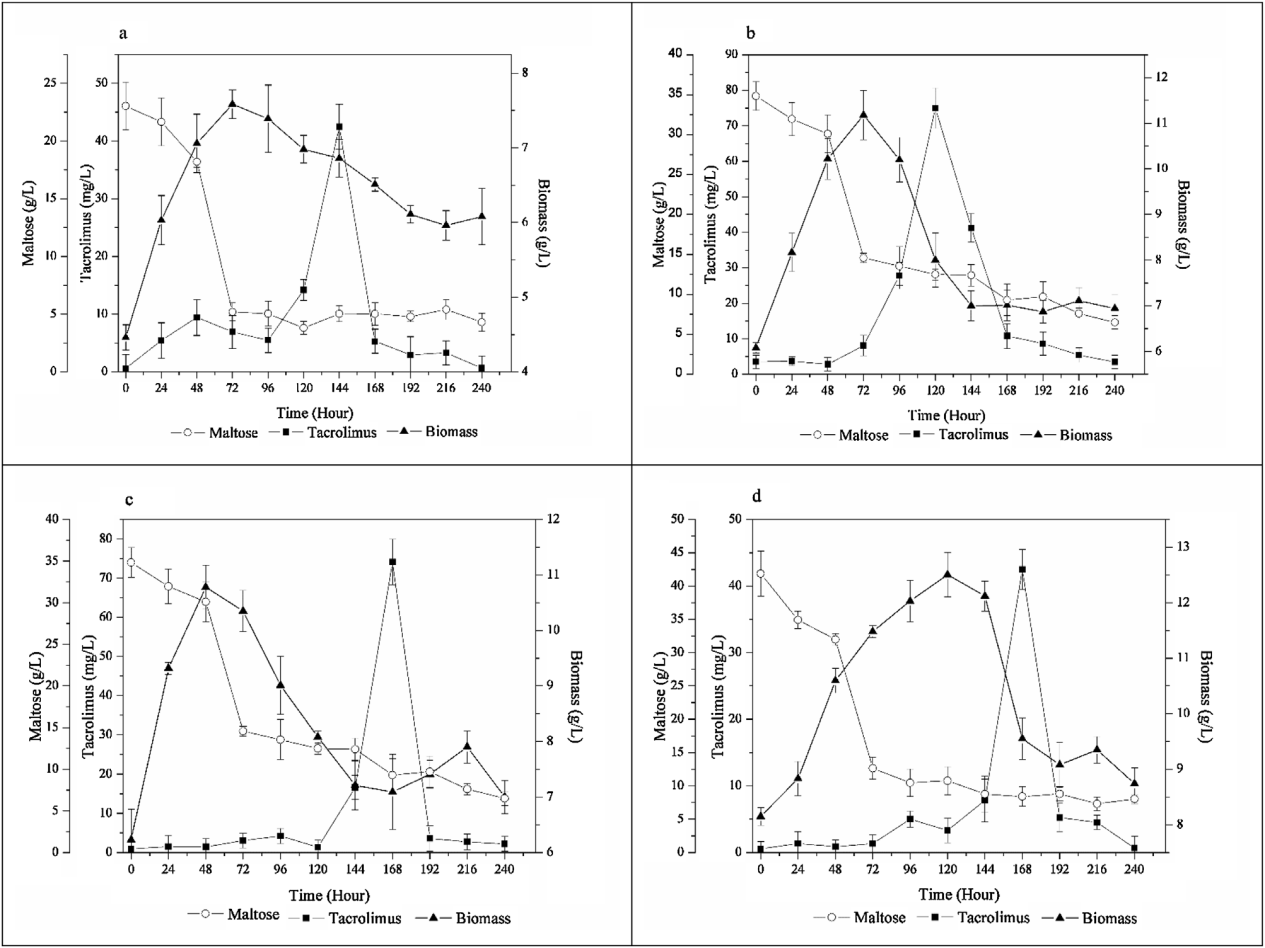
Influence of C:N ratios on tacrolimus production, bacterial growth and maltose consumption (a) C:N ratio 5.9:1.0, (b) C:N ratio 7.2:1.0, (c) C:N ratio 8.5:1.0, (d) C:N ratio 9.8:1.0.

Maltose-containing media were also evaluated by altering the concentration of the primary carbon source, leading to C:N ratios reported in Table 2. The results concerning tacrolimus production, biomass accumulation and maltose consumption during the fermentative process are summarized on Fig. 4.

The time of maximum tacrolimus biosynthesis did not present any clear linearity with the increasing of maltose content on media. However, when we compare the results presented with glucose (Fig. 2) the time of maximum biosynthesis is practically the same, with the exception of glucose at C:N of 9.8;1.0 (Fig. 2d), in which glucose supports a maximum biosynthesis at the shortest time of all experiments.

**Fig. 5:**
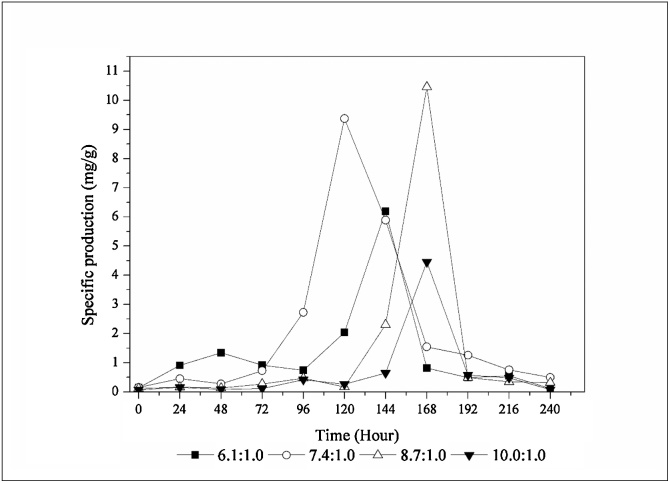
Specific production of tacrolimus with maltose.

Comparing the average results summarized on Fig. 2, Fig. 4, it is readily seen that the MPL media presented higher tacrolimus titer than GPL media, with the exception for the 4 % condition (Figs. 2d and 4 d). However, the most suitable way to compare media is by the specific production plot. Fig. 5 exhibits the specific production of tacrolimus with variation on maltose content on each medium. Comparing the highest specifics productions achieved on this work, when maltose was used as carbon source the fermentation presented 74.15 mg/L of tacrolimus and 7.09 g/L of biomass, resulting in a specific production of 10.46 mg/g (Fig. 4c). When glucose was the primary carbon source, specific production was 9.06 mg/g with 58.74 mg/L of tacrolimus and 6.49 g/L of biomass (Fig. 2c). It can be readily seen that maltose was more supportive to tacrolimus biosynthesis, but it also increased biomass accumulation, leading to a slight increase on specific productivity.

Authors that also used maltose as primary carbon source achieved the specific productivity of 2.5 mg/g [10], 6.0 mg/g [23], and 10.9 mg/g [11]. Turło et al. [10] and Gajzlerska et al. [23] set the process parameters at 30 °C and 110 rpm, while Mishra and Verma [11] set them at 28 °C and 200 rpm. In comparison with the specific production presented in this work, 10. 46 mg/g, process parameters set to the fermentation are of great importance, since they control the kinetics of growth and the aeration of the medium.

### 3.3 Effect of glucose and maltose on tacrolimus specific production

**Fig. 6:**
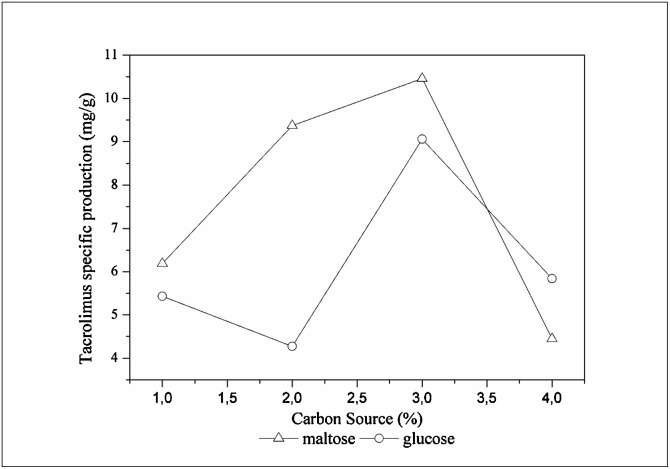
Carbon source dependent profile of tacrolimus specific production by *S. tsukubaensis.*

Fig. 6 exhibits the glucose and maltose dependent profile of tacrolimus specific production at the highest tacrolimus titer of each medium. It demonstrates that maltose-containing media presented an increasing tendency in the specific production up to 3 % of maltose as carbon source in the medium. When glucose was used as carbon source with 1 % and 2 % tacrolimus specific production presented a linear tendency, with a slight decrease, the best results were achieved with 3 % of glucose in the media. For both maltose and glucose, 3 % was the limit of carbon source in the medium without any carbon catabolite repression observed. Media containing 4 % of carbon source presented a sharp downward trend. Chen et al. [13] conducted a series of studies varying the glucose percentage on the medium, the results obtained by authors showed that the increasing of glucose content was beneficial up to 1.75 %, depending on the strain used by the authors.

## 4 Conclusion

The range of carbon content on each medium evaluated on this study presented an increasing tendency on tacrolimus specific productivity up to 3 %. With 4 % of carbon source on medium, tacrolimus biosynthesis presented a sharp downward trend both for glucose and maltose. It indicates that in this study, up to 3 % of carbon source on media does not exert carbon repression, neither by glucose nor by maltose. This work also highlights that process parameters are of great importance on tacrolimus biosynthesis, specially temperature and agitation but also the media composition and genetic enhancement of the strains used for tacrolimus biosynthesis, due to the differences observed with results previously published by other researchers. Our study is of great interest for pharmaceutical industries due to its exploratory characteristic on evaluation of media for submerged fermentation for tacrolimus production. This article highlights the differences between two rapidly consumed carbon sources, glucose and maltose, and the behavior of each one on supporting cell growth and tacrolimus production. Notably, comparing media containing maltose and glucose, it can be readily seen that maltose presented a higher tacrolimus volumetric production. However, when specific productivity is compared, only a slight increase was observed, indicating that maltose not only supports higher tacrolimus biosynthesis but also biomass accumulation.

## CRediT authorship contribution statement

**Jean Vinícius Moreira:** Conceptualization, Methodology, Validation, Investigation. **Seforah Carolina Marques Silva:** Writing - review & editing. **Marco Aurélio Cremasco:** Project administration, Supervision.

## Declaration of Competing Interest

None.
